# Mapping and validation of a novel major QTL for resistance to stripe rust in four wheat populations derived from landrace Qishanmai

**DOI:** 10.3389/fpls.2023.1207764

**Published:** 2023-06-16

**Authors:** Xu Jiang, Zhen Wang, Jing Feng, Ziyi Du, Zhongjun Zhang, Yibin Zhang, Mingzhe Che, Junda Ren, Haiguang Wang, Wei Quan

**Affiliations:** ^1^ Department of Plant Pathology, China Agricultural University, Beijing, China; ^2^ Liaoning Academy of Forestry Sciences, Liaoning Academy of Agricultural Sciences, Shenyang, China; ^3^ Institute of Plant Protection, Chinese Academy of Agricultural Sciences, Beijing, China; ^4^ Open University of China, Beijing, China; ^5^ Institute of Cotton Research, Chinese Academy of Agricultural Sciences, Anyang, China; ^6^ Beijing University of Agriculture, Beijing, China; ^7^ Institute of Hybrid Wheat, Beijing Academy of Agricultural and Forestry Sciences, Beijing, China

**Keywords:** wheat landrace, stripe rust, disease resistance, QTL mapping, marker-assisted selection, QTL interaction

## Abstract

Wheat yield has been constrained by stripe rust disease globally. A wheat landrace (Qishanmai, QSM) consistently showed lower stripe rust severities in multiple year studies than susceptible check varieties including Suwon11 (SW) at the adult plant stage. To detect QTL for reducing the severity in QSM, 1218 recombinant inbred lines (RILs) were developed from SW × QSM. QTL detection was conducted firstly using 112 RILs selected for similarity in pheno-morphological characters. The 112 RILs were assessed for stripe rust severity at the 2nd leaf, 6th leaf and flag leaf stages under field and greenhouse conditions, and genotyping was done primarily with a single nucleotide polymorphism (SNP) array. On the basis of these phenotypic and genotypic data, a major QTL (*QYr.cau-1DL*) was detected on chromosome 1D at the 6th leaf and flag leaf stages. Further mapping was conducted by genotyping 1218 RILs using new simple sequence repeat (SSR) markers, which were developed by referring to the sequences of the wheat line Chinese Spring (IWGSC RefSeq v1.0). *QYr.cau-1DL* was mapped within a 0.5 cM (5.2 Mb) interval delimited by the SSR markers 1D-320.58 and 1D-325.79. These markers were applied to select for *QYr.cau-1DL* by screening F_2_ or BC_4_F_2_ plants of the wheat crosses RL6058 × QSM, Lantian10 × QSM and Yannong21 × QSM. F_2:3_ or BC_4_F_2:3_ families derived from the selected plants were assessed for stripe rust resistance in the fields of two locations and in a greenhouse. Wheat plants carrying the resistant marker haplotype in homozygous state for *QYr.cau-1DL* showed lower stripe rust severities (by 44% to 48%) than plants lacking this QTL. The trial of RL6058 (a carrier of *Yr18*) × QSM also indicated that *QYr.cau-1DL* had larger effect than *Yr18* on reducing severity; they acted synergistically, yielding an elevated level of stripe rust resistance.

## 1 Introduction

Wheat (*Triticum aestivum* L.) is one of the most important staple food crops globally (IWGSC, 2018). Wheat yields have been constrained in many countries/regions of the world by stripe/yellow rust disease caused by the obligate biotrophic fungus *Puccinia striiformis* Westend. f. sp. *tritici* Erikss. (*Pst*). Major *Pst* epidemics have been documented in certain geographical regions of, such as, Australia, China, Europe and the United States of America, causing regional yield losses up to 25% (Wellings, 2011).

Identification and deployment of genes and quantitative trait locus/loci (QTL) conferring resistance to stripe rust is a cost-effective strategy to manage this disease (Johnson, 1984; Line, 2002). Eighty four stripe rust resistance (*Yr*) genes have been formally named (McIntosh et al., 2020; Klymiuk et al., 2022) and more than 350 stripe rust resistance QTL have been documented (Rosewarne et al., 2013; Maccaferri et al., 2015; Jan et al., 2021). During the last two decades, new *Pst* races became prevalent or even predominant in *Pst* populations in some countries/regions, which are virulent to many of the *Yr* genes (Chen, 2005; Milus et al., 2008; Milus et al., 2009; Wellings, 2011; Hovmøller et al., 2016; Ali et al., 2017; Dong et al., 2017) and can also defeat the resistance conferred by certain QTL (Sorensen et al., 2014). Identification of new resistance genes/QTL is an essential work to enhance genetic diversity of resistance resources for sustainably controlling stripe rust.

Some of stripe rust resistance genes/QTL function at all plant growth stages, and some others confer adult plant resistance (APR) (Johnson, 1984; Line, 2002; Dong et al., 2017). The former are usually effective against a limited number of *Pst* races and typically encode NLR proteins (Dong et al., 2017; Javier and Keller, 2021), whereas the latter encode more diverse proteins (Fu et al., 2009; Krattinger et al., 2009; Moore et al., 2015; Dong et al., 2017). APR genes/QTL are often considered to be race non-specific, while exceptions were reported (Johnson, 1984; Johnson, 1992; Singh et al., 2011; Sorensen et al., 2014; Brown, 2015). Certain APR genes/QTL, such as *Yr18*, *Yr29* and *Yr46* (Rosewarne et al., 2013) and *QYrst.wgp-6BS.1*/*Yr78* (Santra et al., 2008; Dong et al., 2017), have been demonstrated to confer durable resistance *sensu* Johnson (1984) and Line (2002). There are also reports where APR was detectable as early as at the 4th leaf stage (Ma and Singh, 1996; Quan et al., 2013; Segovia et al., 2014; Chhetri et al., 2016; Kanwal et al., 2021), being described as “mid-stage resistance” (Kanwal et al., 2021) and “juvenile stage” resistance (Segovia et al., 2014).

Effect size/magnitude of a QTL can be measured in percentage of phenotypic variance explained (PVE%) by the QTL. APR QTL may vary considerably in effect size with a PVE% value ranging from 1% to 88% (Rosewarne et al., 2013; Maccaferri et al., 2015; Jan et al., 2021). QTL with large effects, so-called “major QTL”, are usually more appreciated than minor ones in practical breeding programs (Rosewarne et al., 2013; Wang et al., 2019).

A wheat landrace (Qishanmai, QSM) was observed to possess APR to stripe rust in our previous multiple year studies (**Supplementary Table 1**), although it was susceptible at the seedling stage. The current study was carried out to (1) map QTL for stripe rust resistance in QSM, (2) screen for DNA markers closely flanking a mapped major QTL, (3) validate the major QTL across different environments and genetic backgrounds, and (4) examine combination effect of the mapped QTL with *Yr18*.

## 2 Materials and methods

### 2.1 Mapping population

The mapping population comprising 1218 F_8_ recombinant inbred lines (RILs) from the wheat cross Suwon11 (SW) × QSM was developed as described in Zhang et al. (2023). Briefly, F_2_ was advanced to F_6_ using single seed descent method; seeds of the same RIL were pooled at each of the generations F_7_, F_8_, etc. The winter wheats QSM and SW have the accession numbers S-00402 and AUS-22519, respectively, at http://wheatpedigree.net/. SW is pheno-morphologically similar with QSM as well as is highly susceptible to stripe rust. The wheat variety Mingxian169 (MX) was used as susceptible check throughout the study. QTL mapping was performed firstly using 112 RILs and then the location of a QTL was refined using 1218 RILs.

### 2.2 Preliminary mapping of QTL

#### 2.2.1 Selection of 112 RILs from SW × QSM and disease assessment

Our pilot trials indicated that expression level of the stripe rust resistance in QSM varied with plant growth stages. Here, the resistance of wheat plants was examined at the 2nd leaf, 6th leaf and flag leaf stages. To minimize possible interference with disease recording by difference in growth progress among plants, 112 RILs were selected from the SW × QSM population to serve as test materials for preliminary mapping. The 112 RILs unfolded their main shoot leaves on the same day at each of the 3 plant growth stages and they are also very similar one another in plant height. These RILs together with their parents were evaluated for disease severity in the winter wheat cropping season from autumn 2018 to summer 2019 (abbreviated as “season 2019” hereafter) in the fields of southern Gansu province and Shandong province, and in a greenhouse. Each of the 3 locations was considered as an individual “environment”.

In southern Gansu, trial plots were located in Wushan county (34°42′15′′N, 104°40′08″E, and elevation 1650 m). The trial was carried out in the same ways as in Zhang et al. (2021). Briefly, each plot was constituted with a single 1-m-long row spaced 25 cm apart. A plot was sown with about 40 seeds from an individual RIL of SW × QSM. SW, QSM and the susceptible check wheat MX were grown after every 28 RIL rows. Each RIL was replicated in three plots in a randomized complete block layout. MX was also sown in the bordering areas surrounding the trial plots. As detailed in Zhang et al. (2017) and Wang et al. (2019), the geo-morphological and weather conditions are highly favorable for *Pst* to over-summer, and consequently, to infect the autumn-sown wheat seedlings naturally with no need for artificial inoculation. *Pst* hyphae in the infected wheat plants can over-winter, and then produce and release urediniospores that infect wheat plants in the forthcoming spring (Chen et al., 2009). To minimize a possible cold damage to the wheat plants that harbor *Pst*, the test plots were protected using plastic films in winter months. On drought days in autumn, spring and the early-summer, the test wheat plants were frequently atomized with water after sunset to facilitate *Pst* infection as we previously detailed (Wang et al., 2019; Zhang et al., 2021). *Pst* samples collected from infected MX plants were differentiated for their pathotypes applying the methods used in the USA (Wan and Chen, 2014), but choosing 17 *Yr* genes as differentials that have high differential ability in China (**Supplementary Table 2**). The identified pathotypes were collectively virulent against the genes *1*, *6*, *7*, *8*, *9*, *10*, *17*, *24*, *26*, *27*, *28*, *29*, *32*, *SP* and *Tr1*, and avirulent to *Yr5* and *Yr15* (**Supplementary Table 2**). Disease severity was scored as detailed in Zhang et al. (2017) and Wang et al. (2019). Briefly, the score was in a percentage of infected leaf area (Peterson et al., 1948); stripe rust areas were visually averaged over all the flag leaves within a plot when severities on SW and MX flag leaves reached approximately 90%. Mean severity of three plot replicates for each RIL was used for QTL mapping.

In Shandong, trial plots were located in Tai’an district (36°18′09′′N, 117°13′05″E, and elevation 90 m). The trial was done in the same ways as mentioned above with some exceptions, i.e., spreader (MX) plants were grown in rows adjacent and perpendicular to the test plot rows. Artificial inoculation is needed in this location. The inoculums were the mixed urediniospores of the Chinese *Pst* races CYR32 and CYR34, which have been prevalent in China for multiple years (Han et al., 2015; Wu et al., 2016; Bai et al., 2018). These races are collectively virulent against the *Yr* genes *1*, *2*, *3a*, *4a*, *4b*, *6*, *7*, *8*, *9*, *10*, *17*, *23*, *24*, *25*, *26*, *27*, *28*, *29*, *31*, *43*, *44*, *A*, *Alba*, *Cle*, *CV1*, *CV2*, *CV3*, *Exp2*, *Gaby*, *Res*, *SD*, *SO*, *SP*, *SpP* and *Su*, and avirulent to *Yr5* and *Yr15* (Wan et al., 2004; Li et al., 2006; Chen et al., 2009; Wu et al., 2016). Initial urediniospores of the races were kindly provided by Chen et al. (2009) and Dr. Ruiming Lin (Institute of Plant Protection, Chinese Academy of Agricultural Science). Propagation of urediniospores and inoculation were performed as detailed in Wang et al. (2019) and Zhang et al. (2021). Briefly, the urediniospores were increased on MX plants grown in a greenhouse, and fresh urediniospores were harvested just before they were needed for inoculating the field trial plants. At the tillering to stem elongation stages (mid-March to early April) of the field test plants, a clear afternoon was chosen for inoculation. The MX spreader plants were sprayed with water solution containing well suspended fresh *Pst* urediniospores (in a mean density of about 75,800 urediniospores mL^-1^) and 0.04% Tween 20. Efforts were made to distribute spore dewdrops evenly over wheat leaf surfaces as fine as possible with minimized coalescence; the plants were then protected with plastic films for 16 h. Such an inoculation was repeated three times in the period from mid-March to early April. Three weeks after inoculation, fresh *Pst* urediniospores released from the spreader plants were readily observed. Beginning from this time until last disease recording, the test plants were regularly sprayed with water after sunset. Disease severities were assessed in the ways as mentioned above.

In greenhouse, trial was done in the same ways as described above with some exceptions, i.e., each test plot (20 cm × 20 cm) was sown with 9 seeds from one of the RILs/parents in a drill manner. When their 2nd leaves unfolded completely, the wheat seedlings were inoculated with *Pst* in the same ways as described above. The inoculated seedlings were incubated for 18 h at 6°C to 12°C. The greenhouse temperature was then controlled to a range between 5°C and 25°C with natural daylight supplemented artificially to 16 h at 11000 lux until disease recording was completed. Such a procedure involving inoculation, incubation and greenhouse condition control was repeated when the main shoots’ 6th leaves of the wheat plants unfolded completely (concurrent with 1 to 4 tillers depending on plants). Disease severity was recorded on the inoculated 2nd and 6th leaves, respectively, in the same ways as mentioned above.

#### 2.2.2 Genotyping of the 112 RILs and QTL mapping

Genotyping and QTL mapping were conducted in the same ways as in Zhang et al. (2023). Briefly, DNA samples were prepared from a single F_8_ plant of each of the 112 SW × QSM RILs as well as SW and QSM with the CTAB method in Saghaimaroof et al. (1984). These plants were subjected to a genotypic test applying a 90 K wheat SNP array (Wang et al., 2014) at the CapitalBio Technology (Beijing, China; http://www.capitalbiotech.com). After excluding heterozygous data points, 10026 polymorphic SNPs between SW and QSM were identified from the remaining data. To improve quality, filtering was conducted by removing the SNPs with an allele frequency of lower than 0.35 or higher than 0.65, or with missing scores higher than 5%; 8637 SNPs with high quality were identified. Redundant SNPs were filtered out applying the software in Li et al. (2007) and manually; the retained 1578 unique SNP markers were subjected to a linkage analysis applying JoinMap 4.0 (Stam, 1993). Genetic distance (in cM) was calculated using Kosambi function. Each linkage group was assigned to a specific chromosome by aligning the sequences containing the SNPs to the chromosomal survey sequence map (IWGSC, 2014; IWGSC, 2018).

However, we observed that the SNP map contained 32 large gaps (>20 cM). To reduce such gaps, 988 SSR markers were developed on the basis of the sequence IWGSC RefSeq v1.0 (IWGSC, 2018). These markers were designated with chromosome name plus numbers indicating coordinates in Mb in the IWGSC RefSeq v1.0; for instance, “1D-322.55” denotes an SSR marker physically positioned at 322.55 Mb on chromosome 1D. PCR reaction was carried out as we previously described (Zhang et al., 2023). Briefly, each reaction tube included 4.65 μL ddH_2_O, 10 × PCR buffer (Mg^2+^ plus, 1.0 μL), DNA template (25–55 ng/μL, 2.0 μL), forward and reverse primers (both in 10 μM, 1.0 μL each primer), dNTPs (10 mM, 0.25 μL), and Taq DNA polymerase (5 unit/μL, 0.1 μL). PCR amplification was performed following Roder et al. (1998), i.e., started with 94°C for 3 min, then 45 cycles at 94°C for 1 min, 60°C for 1 min and 72°C for 2 min, and ended with 72°C for 10 min. In cases where this cycling failed to produce amplicons, a touchdown program was used, i.e., started with 94°C for 5 min; then 10 cycles at 94°C for 30 s, 60°C for 30 s (touchdown with -0.5°C each cycle), and 72°C for 30 s; followed by 35 cycles at 94°C for 30 s, 55°C for 30 s, and 72°C for 30 s; and ended with 72°C for 10 min. PCR products were differentiated using 6% denaturing polyacrylamide gels and stained with AgNO_3_ (Bassam et al., 1991). Thirty-two of the 988 markers were selected to genotype the 112 RILs. Sequences and details of the 32 PCR markers including the PCR profiles were presented in **Supplementary Table 3**.

The composite interval mapping (CIM) method in Windows QTL Cartographer 2.5 (Wang et al., 2010) was chosen to do QTL mapping. A threshold LOD value was determined for each of the trials applying the permutation program that was run repeatedly for 1000 times using *α* = 0.05 as the type I error rate. The threshold value varied with different trials, ranging from 2.7 to 3.5. For the sake of simplicity, 3.5 was used as the threshold for all trials. CIM control parameters were set with the model 6 (standard model), a window size of 10 cM, and the forward and backward regression at a criteria probability of 0.05 for both “into” and “out”. The walking speed was at 0.5 cM. Effect size/magnitude of a QTL was expressed in phenotypic variance explained (PVE) by the QTL.

### 2.3 Further mapping of a detected QTL

A major QTL, detected using the aforementioned 112 RILs, was further mapped using all the 1218 RILs and hundreds of new SSR markers that were developed and named as mentioned above. These markers were screened firstly against SW and QSM and then against SW × QSM RILs. It was found that two (1D-298.34 and 1D-356.22) (**Supplementary Table 3**) of these markers delimited the detected major QTL to a relatively short interval. From the 1218 RILs, 92 recombinants were identified between 1D-298.34 and 1D-356.22. The 92 RILs were assessed for stripe rust resistance in season 2020 in the fields of southern Gansu and Shandong, and in a greenhouse as described above.

### 2.4 Validation populations and disease assessments

To validate a QTL mapped in SW × QSM population, three wheat populations were used, namely, 30 BC_4_F_2:3_ families selected from Lantian10 (LT) × QSM, 30 BC_4_F_2:3_ families from Yannong21 (YN) × QSM, and 80 F_2:3_ families from RL6058 × QSM. Such selections were conducted using DNA markers that tag the mapped QTL as detailed in **Supplementary File 1**. LT and YN were commercial cultivars, respectively, in southern Gansu and Shandong with susceptibility to stripe rust. RL6058 carries *Yr18*, and thus RL6058 × QSM was also used for examining interaction between the detected QTL and *Yr18*. The plants of LT × QSM, YN × QSM, and RL6058 × QSM were tested in season 2022 for stripe rust resistance in, respectively, southern Gansu, Shandong, and a greenhouse. The trials were conducted basically in the same ways as described above, but seeds were sown individually 20 cm apart. Greenhouse conditions were the same as mentioned above. Disease severities were assessed on flag leaves of individual plants in all the three locations. Infection types were also recorded in southern Gansu based on a 1 to 9 scale following Line and Qayoum (1992).

### 2.5 Statistical analyses of disease data

Disease data were analyzed applying SAS/STAT version 9.3 (SAS Institute Inc., Cary, NC, USA). Analyses of variance, heritability and correlation for RIL disease data were performed with PROC GLM, PROC VARCOMP and PROC CORR, respectively. Disease means between different groups of BC_4_F_2:3_ or F_2:3_ plants were compared based on ANOVA and Tukey test at α = 0.05.

## 3 Results

### 3.1 Mapping of QTL using 112 RILs of SW × QSM

**Figure 1:**
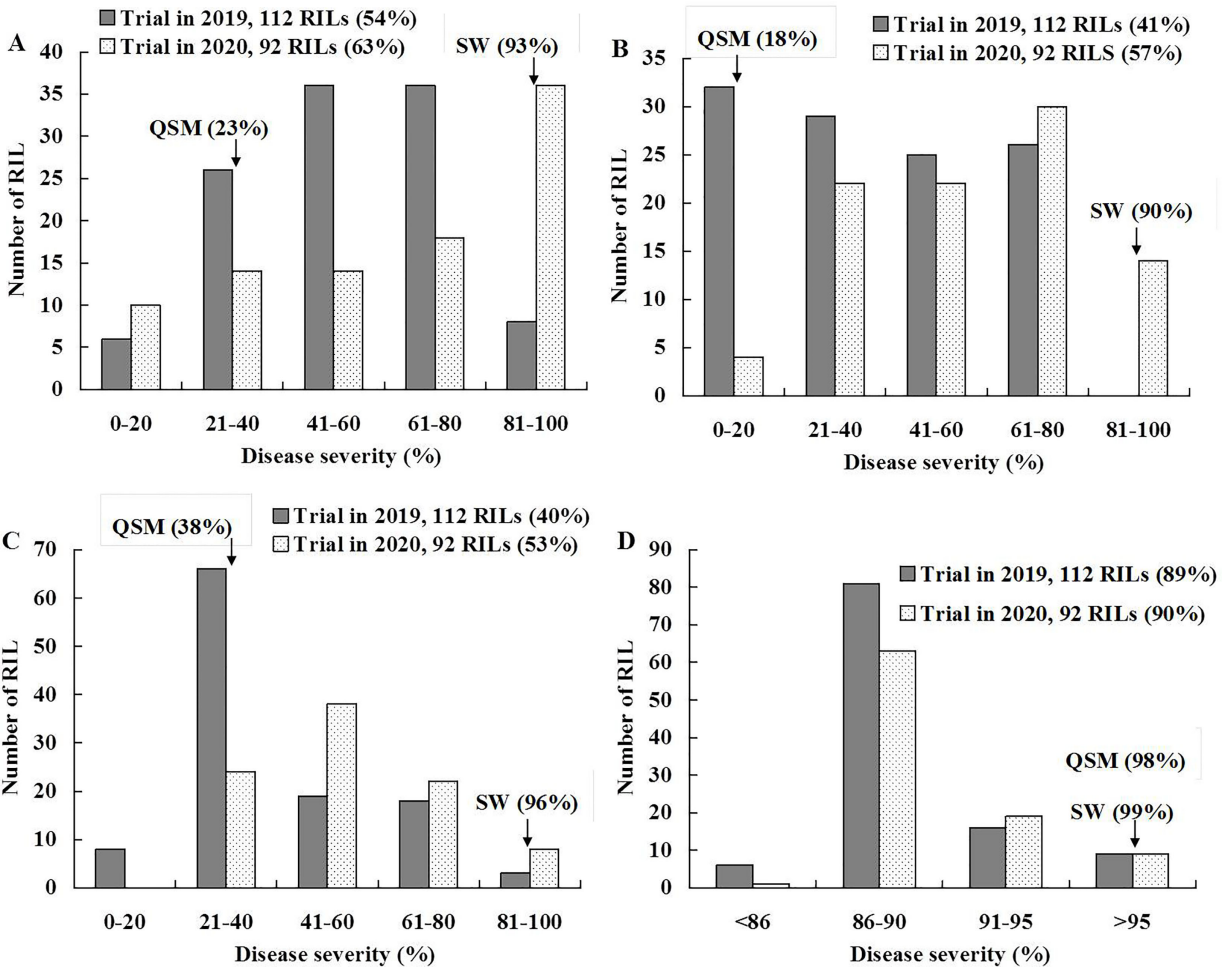
Frequency distribution of recombinant inbred lines (RILs) of Suwon11 (SW) × Qishanmai (QSM) for stripe rust severity. Disease was recorded on flag leaves in the fields of southern Gansu province **(A)** and Shandong province **(B)**, and on 6th leaves **(C)** and 2nd leaves **(D)** in greenhouses. Mean severities are presented in parentheses.

Stripe rust severities were equal to or higher than 90% on the susceptible parent SW at all stages and on the APR parent QSM at the 2nd leaf stage (**Figure 1**; **Supplementary File 2** and **Supplementary Table 4**). At the 6th leaf and flag leaf stages, however, QSM showed obviously lower severities than SW, confirming our previous observation that QSM possesses APR. The disease severities of the 112 RILs ranged broadly from 5% to 100% (**Figures 1A–C**) and varied significantly among the RILs at the 6th leaf and flag leaf stages (**Table 1**), compared to the narrow range (**Figure 1D**) and insignificant variance at the 2nd leaf stage (**Table 1**). Correlation coefficients for RIL disease severity were significant between different plant growth stages and among different test environments (**Table 2**) with a heritability being 0.89. These data illustrate that the APR was genetically segregating in a quantitative manner and the resistance began to express at the 6th leaf stage.

**Table 1:** Analysis of variance on disease severity of the recombinant inbred lines (RILs) of Suwon11 (SW) × Qishanmai (QSM).

Growth stage	Test environment	Source of variance	*df*	MS	*F*	*P*	*df*	MS	*F*	*P*
			112 RILs^a^ tested in 2019				92 recombinants^b^ in 2020			
Flag leaf	Fields in Gansu	RILs	111	1169	29.2	<0.0001	91	2109	55.4	<0.0001
		Replicates	2	5	0.1	0.8929	2	33	0.9	0.4196
Flag leaf	Fields in Shandong	RILs	111	1324	17.5	<0.0001	91	1518	21.0	<0.0001
		Replicates	2	286	3.8	0.0244	2	299	4.2	0.0174
6th leaf	Greenhouse	RILs	111	836	9.2	<0.0001	91	1057	25.3	<0.0001
		Replicates	2	359	4.0	0.0202	2	96	2.3	0.1042
2nd leaf	Greenhouse	RILs	111	23	0.8	0.9029	91	21	0.7	0.9703
		Replicates	2	54	1.9	0.1480	2	83	2.7	0.0677

a The 112 RILs were selected from the entire SW × QSM population (1218 RILs) for similarity in pheno-morphological characters

b The 92 recombinants were identified by screening the 1218 RILs based on the SSR markers 1D-298.34 and 1D-356.22 as shown in **Figure 2B**.

**Table 2:** Correlation coefficients for disease severity of recombinant inbred lines (RILs) of Suwon11 × Qishanmai among different plant growth stages and test environments.

Plant growth stage, Test environment	Flag leaf, Shandong field	6th leaf, Greenhouse	Flag leaf, Shandong field	6th leaf, Greenhouse
	Selected 112 RILs		Recombinants (92 RILs)	
Flag leaf, Gansu field	0.76^a^	0.72^a^	0.91^a^	0.85^a^
Flag leaf, Shandong field		0.75^a^		0.83^a^

a P<0.0001.

The genetic map of the 112 RILs covers all 21 chromosomes with 1610 (1578 SNP and 32 SSR) markers and spans 3656.4 cM (**Supplementary Table 5**). The mean distance between markers was 2.3 cM with a maximum of 19.2 cM and a minimum of 0.1 cM. Based on this map and disease severity data of the 112 RILs, a QTL (*QYr.cau-1DL*) was detected on chromosome arm 1DL (**Figure 2A**). *QYr.cau-1DL* showed a LOD peak value from 12.4 to 23.4 that exceeded the threshold (3.5) at the 6th leaf and flag leaf stages in all trials (**Table 3**); at the 2nd leaf stage, however, LOD value was lower than 3.5 (**Figure 2A**). *QYr.cau-1DL* was flanked within a 5.7 cM interval by the SNP markers IWB15913 and IWB2332 (**Figure 2A**). IWB12282 was located at the chromosome position where LOD peak of *QYr.cau-1DL* occurred. Particularly, we could identify an SSR marker (1D-322.55) that was also located at the peak (**Figure 2A**). *QYr.cau-1DL* illustrated a PVE value of 35% at the 6th leaf stage and 48% to 55% at the flag leaf stage (**Table 3**); the stripe rust resistant allele was contributed by QSM.

**Figure 2:**
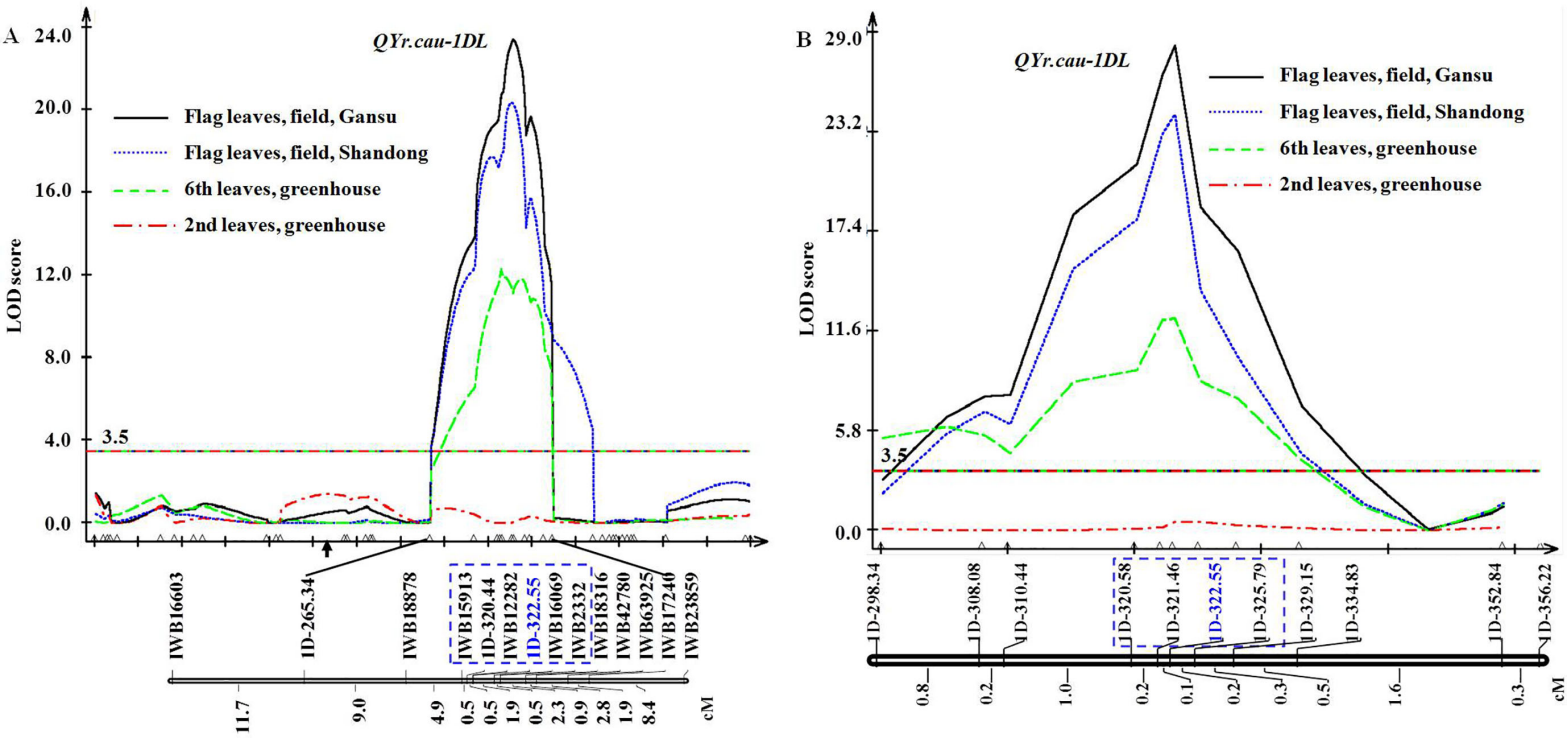
Genetic map of *QYr.cau-1DL* region in chromosome arm 1DL and logarithm of the odds (LOD) curves. **(A)** Location of *QYr.cau-1DL* mapped using 112 recombinant inbred lines (RILs). **(B)** Location of *QYr.cau-1DL* refined based on the map of 1218 RILs. Marker names and intervals (in cM of Kosambi) between adjacent markers are shown along the chromosome orientated with the telomere of 1DL to the right. Arrow suggests approximate position of the centromere. Horizontal lines indicate the threshold LOD of 3.5. Each of the small triangles along the x axis represents a marker used for QTL mapping. For reading ease, only certain marker names are displayed, while all marker names with their positions are presented in **Supplementary Table 5**.

**Table 3:** A QTL (*QYr.cau-1DL*) on chromosome arm 1DL for stripe rust resistance in the recombinant inbred line (RIL) mapping population of Suwon11 (SW) × Qishanmai (QSM), detected in different environments.

Set of RILs /Test environment	Plant growth stage	Flanking markers^a^ (physical position in Mb)^b^	LOD	PVE^c^ (%)
Selected 112 RILs^d^				
/Field, 2019, Gansu	Flag leaf	IWB15913 (318.10), IWB2332 (329.74)	23.4	55
/Field, 2019, Shandong	Flag leaf	IWB15913 (318.10), IWB2332 (329.74)	20.3	48
/Greenhouse, 2019	6th leaf	IWB15913 (318.10), IWB2332 (329.74)	12.4	35
Recombinants (92 RILs)^e^				
/Field, 2020, Gansu	Flag leaf	1D-320.58 (320.58), 1D-325.79 (325.79)	28.5	64
/Field, 2020, Shandong	Flag leaf	1D-320.58 (320.58), 1D-325.79 (325.79)	24.6	56
/Greenhouse, 2020	6th leaf	1D-320.58 (320.58), 1D-325.79 (325.79)	12.5	41

a SSR markers were named using chromosome name followed by coordinates in Mb in IWGSC RefSeq v1.0 (IWGSC, 2018).

b Physical position was inferred by aligning the marker sequences to the sequence in IWGSC RefSeq v1.0 (IWGSC, 2018).

c Phenotypic variance explained by QTL. The stripe rust resistant allele came from Qishanmai.

d The 112 RILs were selected, for similarity in pheno-morphological characters, from the entire SW × QSM population (1218 RILs).

e The recombinants (92 RILs) were identified from the 1218 RILs based on the SSR markers 1D-298.34 and 1D-356.22 that flank QYr.cau-1DL (**Figure 2B**).

Additional QTL were mapped on chromosomes 3A, 5A, 6A and 7A with PVE values being lower than 11%. At two of the four loci, resistant alleles came from QSM. These minor QTL will be further examined in future. Only *QYr.cau-1DL* is addressed here.

### 3.2 Further mapping of *QYr.cau-1DL* using 1218 RILs of SW × QSM

The genetic map (**Figure 2A**) constructed using 112 RILs provided an SSR marker (1D-322.55) that was considered as a tag for *QYr.cau-1DL*. Around the position of 1D-322.55 in the reference physical assembly (IWGSC, 2018), 607 new SSR markers were developed and used firstly to check QSM and SW. The identified 189 polymorphic markers were then applied to screen 20 RILs, sampled randomly from the 1218 ones, for checking marker segregation and quality. From the 189 markers, 18 ones of high quality were selected to genotype all 1218 RILs; 10 of the 18 markers (**Supplementary Table 3**) were mapped around 1D-322.55 (**Figure 2B**). Genetic distance between the most proximal marker (1D-298.34) and the most distal marker (1D-356.22) was 5.2 cM. Ninety-two out of the 1218 RILs were identified to be recombinants (**Supplementary Table 6**) between 1D-298.34 and 1D-356.22. Disease data of the 92 RILs consolidated the disease results of the 112 RILs as shown in **Figure 1** and **Tables 1**, **2**.

Based on the disease severity data of the 92 RILs (**Supplementary Table 6**) and the genetic map of 1218 RILs (**Figure 2B**), *QYr.cau-1DL* was delimited, by 1D-320.58 and 1D-325.79 (**Figure 2B**), to a 0.5 cM genetic interval that was equivalent to a physical distance of 5,207,169 bp on 1D, spanning 320,584,561–325,791,730 bp in the reference map (IWGSC, 2018).

### 3.3 Validation of *QYr.cau-1DL via* marker-based selection

**Figure 3:**
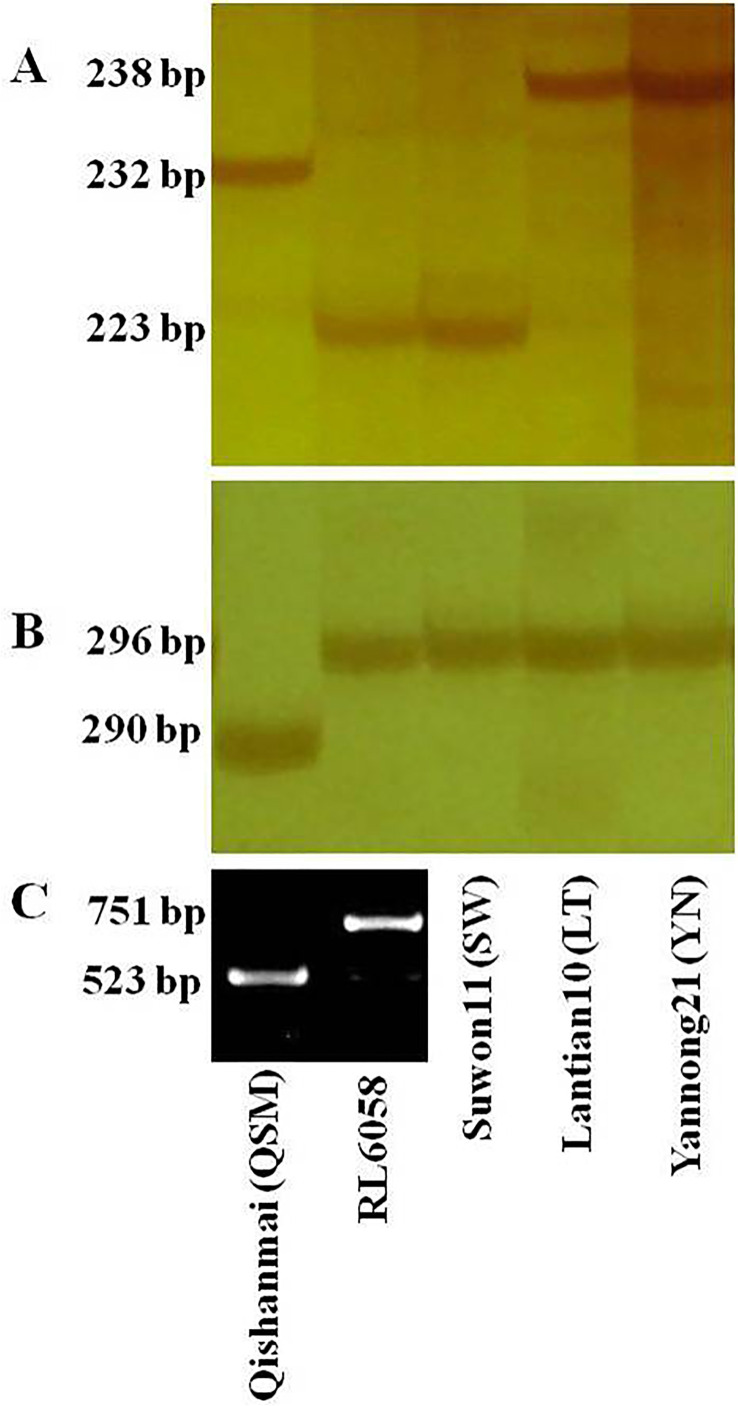
DNA fragments produced from the marker loci 1D-320.58 **(A)** and 1D-325.79 **(B)** for *QYr.cau-1DL*, and *cssfr5* **(C)** for *Yr18* by PCR amplification with DNA templates from the five tested wheat lines. DNA fragments were separated using polyacrylamide gels for **(A, B)**, while using agrose gel for **(C)**. Primer sequences are presented in **Supplementary Table 3**.

PCR amplicon of QSM DNA was a 232 bp fragment at 1D-320.58 and a 290 bp fragment at 1D-325.79 (**Figure 3**); combination of the two fragments constitutes the marker haplotype for the resistant allele of *QYr.cau-1DL*. RL6058 and SW showed a susceptible haplotype, and LT and YN showed another susceptible haplotype. QSM had a 523 bp amplicon that represents the susceptible allele of *Yr18*, while the 751 bp amplicon of RL6058 represents the resistant allele (**Figure 3**).

**Figure 4:**
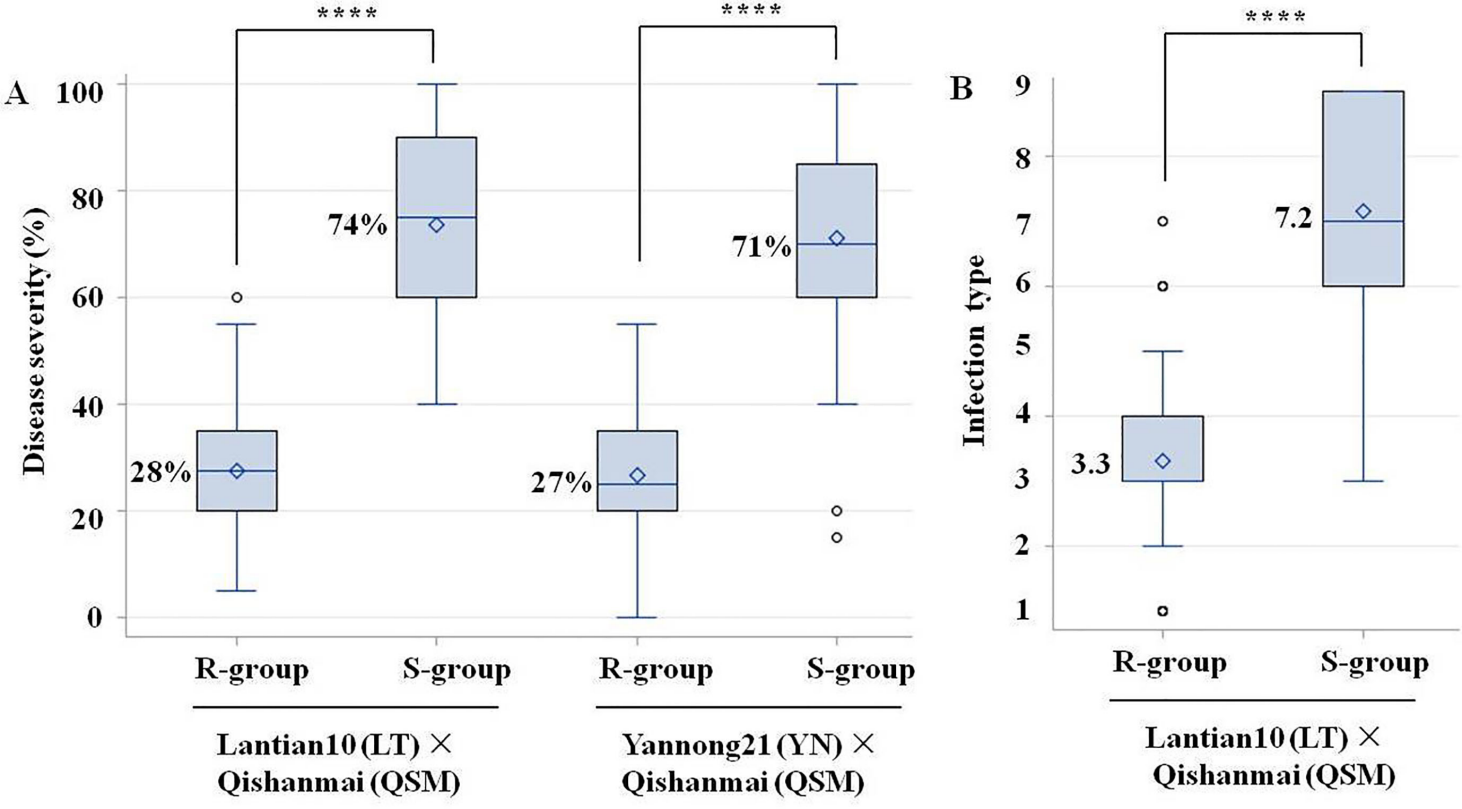
Boxplots showing effects of *QYr.cau-1DL* (represented by the flanking markers 1D-320.58 and 1D-325.79) on reducing stripe rust severity **(A)** and infection type **(B)** in Gansu field for LT × QSM and Shandong field for YN × QSM. R-plants carried the marker haplotype of stripe rust resistance in homozygous state at *QYr.cau.-1DL* and S-plants carried the haplotype of susceptibility in homozygous state. **** Indicates significant difference at α = 0.0001 based on an ANOVA and Tukey test. Small diamond and solid line within a box indicate the mean and median disease value, respectively. The top and bottom edges of a box illustrate the 75th and 25th percentiles, respectively. The whiskers outside a box extend to the extreme data points, and small circles denote outliers.

The R-plants and S-plants of BC_4_F_2:3_ families selected from LT × QSM (**Supplementary File 1**) had a homozygous resistant haplotype at *QYr.cau-1DL* and a homozygous susceptible haplotype, respectively. The R-plants showed a significantly lower mean disease severity (by 46%) than the S-plants (**Figure 4A**; **Supplementary Table 7**). Significant difference was also observed for infection type (**Figure 4B**) between the R- and S-plants. Correlation between disease severity and infection type was positive and significant (*r* = 0.96, *P* < 0.0001). Likewise, comparison between the R- and S-plants selected from YN × QSM (**Supplementary File 1**) showed a lower severity (by 44%) in the former than that in the latter (**Figure 4A**; **Supplementary Table 8**).

**Figure 5:**
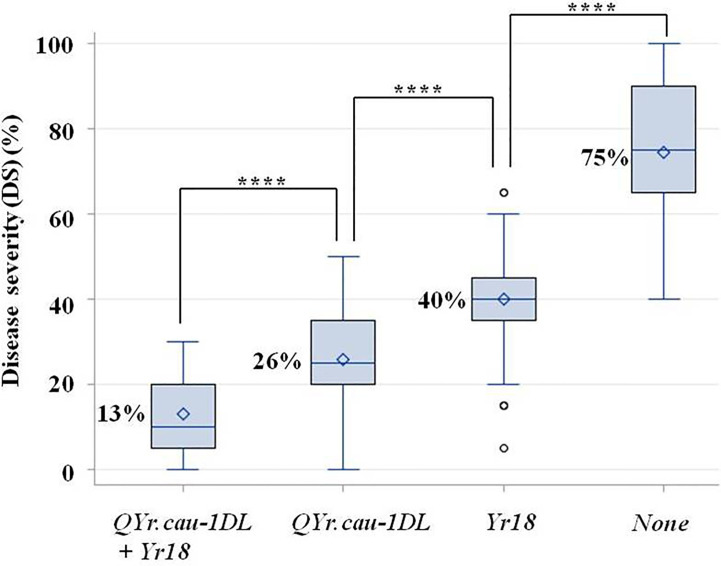
Boxplots showing the effects of QTL combinations on stripe rust severity in RL6058 × Qishanmai F_2:3_ population in a greenhouse. The x-axis defines four F_2:3_ family groups with different QTL combinations, i.e., presence of *QYr.cau-1DL* plus *Yr18*, *QYr.cau-1DL* alone, *Yr18* alone, and *None* (neither *QYr.cau-1DL* nor *Yr18*), respectively. Refer to the legend of **Figure 4** for descriptions of **** and box.

The four groups of F_2:3_ plants selected from RL6058 × QSM, namely, *QYr.cau-1DL*+*Yr18*, *QYr.cau-1DL* alone, *Yr18* alone, and *None* (**Supplementary File 1**; **Figure 5**), were significantly different in disease severity (**Figure 5**; **Supplementary Table 9**). The lowest (13%) and highest (75%) severities were observed in the first group (i.e., *QYr.cau-1DL*+*Yr18*) and in the last group (*None*; susceptible control), respectively. The group carrying *QYr.cau-1DL* alone displayed a lower severity (by 14%) than the group carrying *Yr18* alone, and a further lower severity (by 49%) than the susceptible control group (**Figure 5**).

## 4 Discussion

Wheat landraces have been considered to possess a higher level of genetic diversity than modern cultivars (Newton et al., 2010; Marone et al., 2021). A number of new stripe rust resistance genes/QTL have been found from wheat landraces as exemplified by those in the Watkins Collection (Bansal et al., 2011) and the Vavilov Collection (Jambuthenne et al., 2022). Likewise, the *QYr.cau-1DL* identified here from the landrace QSM constitutes an example from China, where there is a long wheat cultivation history with considerably diverse landraces (Zhou et al., 2018).

It is notable that *QYr.cau-1DL* was significantly effective in all the 9 field and greenhouse trials and in all the 4 wheat crosses SW × QSM, LT × QSM, YN × QSM and RL6058 × QSM (**Table 3**; **Figures 2**, **4**, **5**). The plants selected from RL6058 × QSM have a genetic background of spring habit (**Supplementary File 1**), contrasting to the winter/facultative habit of the other 3 crosses. The semi-dwarf wheat cultivar LT (95 cm) and dwarf YN (75 cm) were substantially different each other in a number of pheno-morphological traits (data not shown) and adapt to highland (Gansu province, elevation 1650 m) and lowland (Shandong province, 90 m) ecosystems, respectively. The trial plots in Gansu were separated by 1100 km from those in Shandong. These data indicate that *QYr.cau-1DL* can consistently act in greatly varied circumstances.

### 4.1 Novelty of *QYr.cau-1DL*

**Figure 6:**
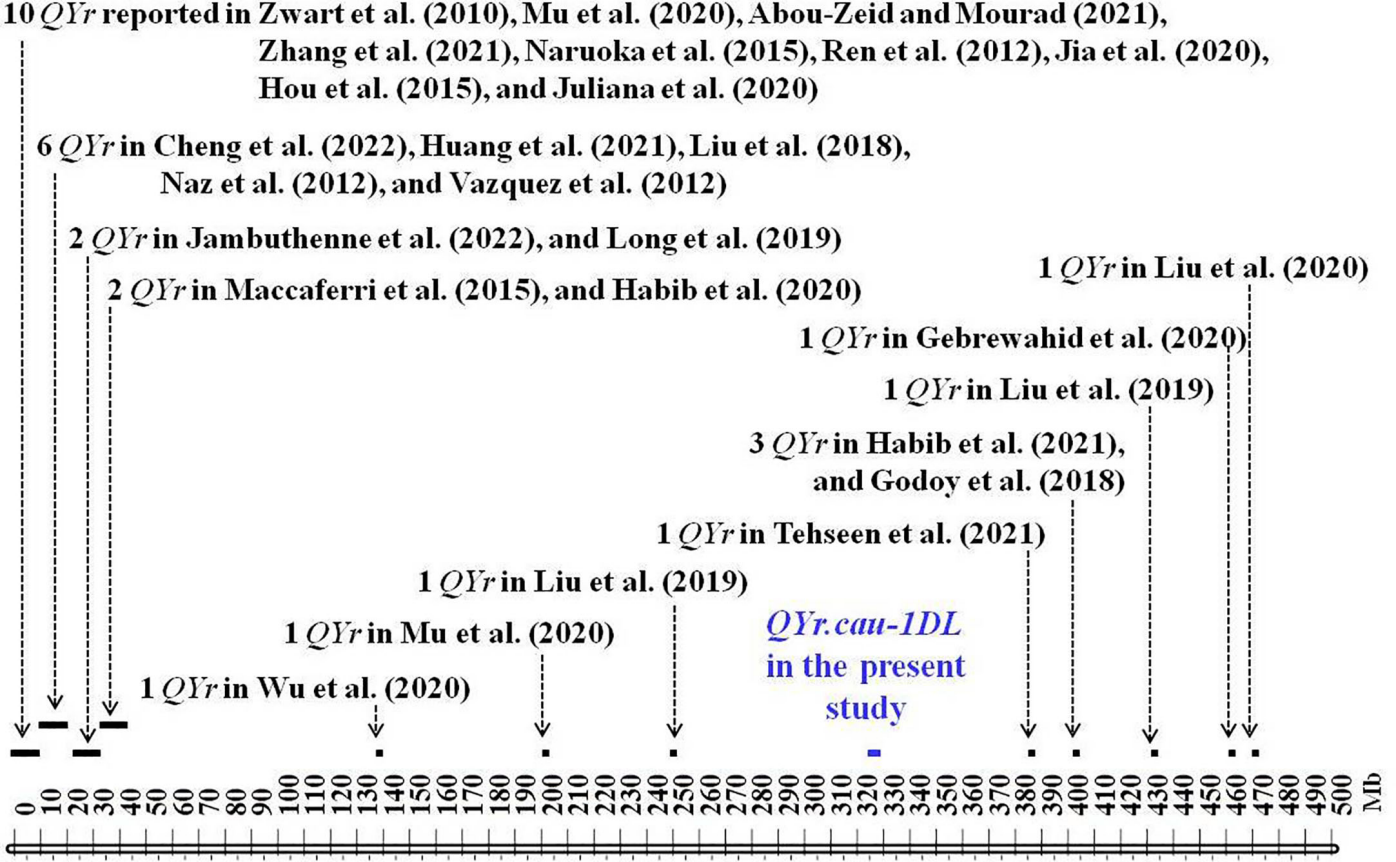
Physical positions of *QYr.cau-1DL* and the previously reported QTL for resistance to stripe rust on chromosome 1D (10-Mb tick size map) inferred by aligning marker sequences of these QTL to the 1D sequences in the IWGSC RefSeq v1.0 (IWGSC, 2018). Arrows indicate the approximate positions of previously reported QTL. Refer to **Supplementary Table 10** for further information and a list of full references.

*QYr.cau-1DL* is located on chromosome 1D where 30 stripe rust resistance QTL were previously detected (**Figure 6**; **Supplementary Table 10**). These QTL were physically projected by aligning the sequences of their representing marker/s to 1D sequence in the IWGSC RefSeq v1.0 (**Supplementary Table 10**). *QYr.cau-1DL* was projected to a 5.2 Mb region, spanning 320.58–325.79 Mb. There is a distance of >60 Mb between *QYr.cau-1DL* and the other 30 QTL (**Figure 6**). *Yr25* was also located on 1D by Calonnec and Johnson (1998) using Chinese Spring monosomic lines, but it could not be physically positioned due to lack of DNA marker. Our observations illustrated that the wheat variety Reichersberg 42 (a carrier of *Yr25*) was highly susceptible (**Supplementary File 2**) in terms of both infection type and disease severity. Therefore, it is highly likely that *QYr.cau-1DL* is a new QTL for resistance to stripe rust.

### 4.2 Combination of *QYr.cau-1DL* with *Yr18*

*Yr18* is an important resistance resource since it confers durable resistance (Dyck et al., 1966; Krattinger et al., 2009). RL6058 carries *Yr18* (Lagudah et al., 2009) and thus RL6058 × QSM F_2:3_ families were segregating at both *QYr.cau-1DL* and *Yr18*. This provides an opportunity for comparing effects of the two QTL in the same genetic background and also for examining their interaction. *QYr.cau-1DL* displayed a larger effect on reducing stripe rust severity than *Yr18* (**Figure 5**). Rosewarne et al. (2013) indicated that “*Yr29* may be less effective against stripe in the presence of *Yr18*”; similar interaction between *Yr18* and *Yr46* was addressed by Lagudah (2011). Different from these situations, combination of *QYr.cau-1DL* with *Yr18* can elevate the level of resistance (**Figure 5**) without unfavorable interaction. The RL6058 × QSM results also illustrate that *QYr.cau-1DL* can be effectively selected based on the resistant marker haplotype, i.e., a 232 bp amplicon at 1D-320.58 combined with a 290 bp amplicon at 1D-325.79 (**Figure 3**). The 751 bp amplicon, a fragment of the cloned *Yr18* gene, is diagnostic of the resistant allele at *Yr18* (Krattinger et al., 2009; Lagudah et al., 2009). Therefore, *QYr.cau-1DL* and *Yr18* can be utilized simultaneously in breeding programs with marker-assisted selection.

### 4.3 Effectiveness of *QYr.cau-1DL* against diverse *Pst* virulent variants

We previously described (Zhang et al., 2017; Wang et al., 2019) that southern Gansu is a *Pst* hotspot within the near-Himalayan region. This region has been hypothesized to be a part of *Pst* diversity center (Ali et al., 2014). We also described some supporting data (Zhang et al., 2017; Wang et al., 2019). In southern Gansu and northwestern Sichuan of China, for instance, the geo-morphological and weather conditions are highly favorable for *Pst* to over-summer, over-winter and survive year-round, leading to a brooder of new *Pst* virulence races (Wan et al., 2004; Chen et al., 2009). *Pst* population can reproduce sexually on alternate hosts with naturally occurring recombinants being observed (Mboup et al., 2009; Ali et al., 2010; Duan et al., 2010; Zhao et al., 2016). Since 1940s when the first survey on *Pst* races was conducted in China (Chen et al., 2009), extraordinarily diverse virulent variants have been identified (Wan et al., 2004; Chen et al., 2009; Bai et al., 2018; Li et al., 2023), and nearly every of *Pst* races first appeared in the hotspot. Resistance genes, as exemplified by *Yr9* and *Yr26*, also first became ineffective there (Wan et al., 2004; Chen et al., 2009; Han et al., 2015; Bai et al., 2018). We consider that any race-specific stripe rust resistance gene/QTL might be defeated by *Pst* variants in such a hotspot within limited years. But, during the 36 years from 1987 to 2022, stripe rust severities never reached 25% on QSM (the donor of *QYr.cau-1DL*), relative to >85% on the susceptible check wheat MX (**Supplementary Table 1**). Another fact is that QSM was cultivated on approximately 43000 hectares annually in the 1940s and early 1950s in southern Gansu (Zhang, 1995), namely, this landrace was ever widely grown. Therefore, *QYr.cau*-*1DL* might be associated with a form of durable resistance *sensu* Johnson (1984).

## 5 Conclusion

An APR QTL (*QYr.cau-1DL*) identified in the present study has larger effects on reducing stripe rust severity than the well-known gene *Yr18*; synergistic interaction between the two QTL can yield an elevated level of resistance. *QYr.cau-1DL* can stably function over substantially diverse genetic backgrounds and highly different agro-geographical environments. Thus, it should have a potential for application in wheat breeding across wide circumstances. The SSR markers 1D-320.58 and 1D-325.79 can be used to assist selection for *QYr.cau-1DL* in breeding processes following parental polymorphism checks.

## Data availability statement

The original contributions presented in the study are included in the article/**Supplementary Material**. Further inquiries can be directed to the corresponding author.

## Ethics statement

The authors declare that the experiments comply with the current laws of the country.

## Author contributions

XJ and WQ conceived the study. JF, ZZ, and WQ constructed the wheat populations. XJ, ZW, ZD, ZZ, WQ, YZ, MC, and JR prepared inoculums, carried out disease trials, and genotyped wheat lines using SSR markers. XJ, ZW, and ZZ designed SSR primers and constructed linkage groups. XJ and ZZ analyzed disease data. HW contributed to inoculums preparation and supervised XJ in learning related to this study. XJ drafted the manuscript. All authors contributed to the article and approved the submitted version.
