# Supplementary material for: Mapping and validation of a novel major QTL for resistance to stripe rust in four wheat populations derived from landrace Qishanmai
Source: Front Plant Sci. 2023 Jun 16;14:1207764. doi: 10.3389/fpls.2023.1207764 (PMC10311914; doi:10.3389/fpls.2023.1207764)

Supplementary Material

Mapping and validation of a novel major QTL for resistance to stripe rust in four wheat populations derived from landrace Qishanmai

Xu Jiang, Zhen Wang, Jing Feng, Zhongjun Zhang, Yibin Zhang, Mingzhe Che, Junda Ren, Haiguang Wang and Wei Quan*

*** Correspondence author**

Wei Quan
quanwei_baafs@sina.com

Supplementary File 1 | Construction of wheat populations to validate a detected QTL for resistance to stripe rust

A QTL (*QYr.cau-1DL*) for resistance to stripe rust was detected in the mapping population of Suwon11 (SW) × Qishanmai (QSM) in the present study. To validate *QYr.cau-1DL* in different genetic backgrounds, three wheat populations were constructed, namely, 30 $BC_4F_{2:3}$ families of Lantian10 (LT) × QSM, 30 $BC_4F_{2:3}$ families of Yannong21 (YN) × QSM, and 80 $F_{2:3}$ families of RL6058 × QSM. LT (pedigree: Xifeng16/76-89-13) and YN (Yan1933/Shan82-29) have been commercially cultivated in China, but they are susceptible to stripe rust. RL6058 (Thatcher*6/PI 58548) carries *Yr18* (Lagudah et al. 2009).

(1) $BC_4F_{2:3}$ families of LT × QSM and YN × QSM

As schematically shown below, QSM was crossed with LT and YN, respectively, in May 2015. Backcrossing (with LT and YN as recurrent parents) and phenotypic selection for stripe rust resistance were conducted during the period from May 2016 to June 2020. BC_4F_2 seeds were harvested from the BC_4F_1 plants that showed resistance to stripe rust.

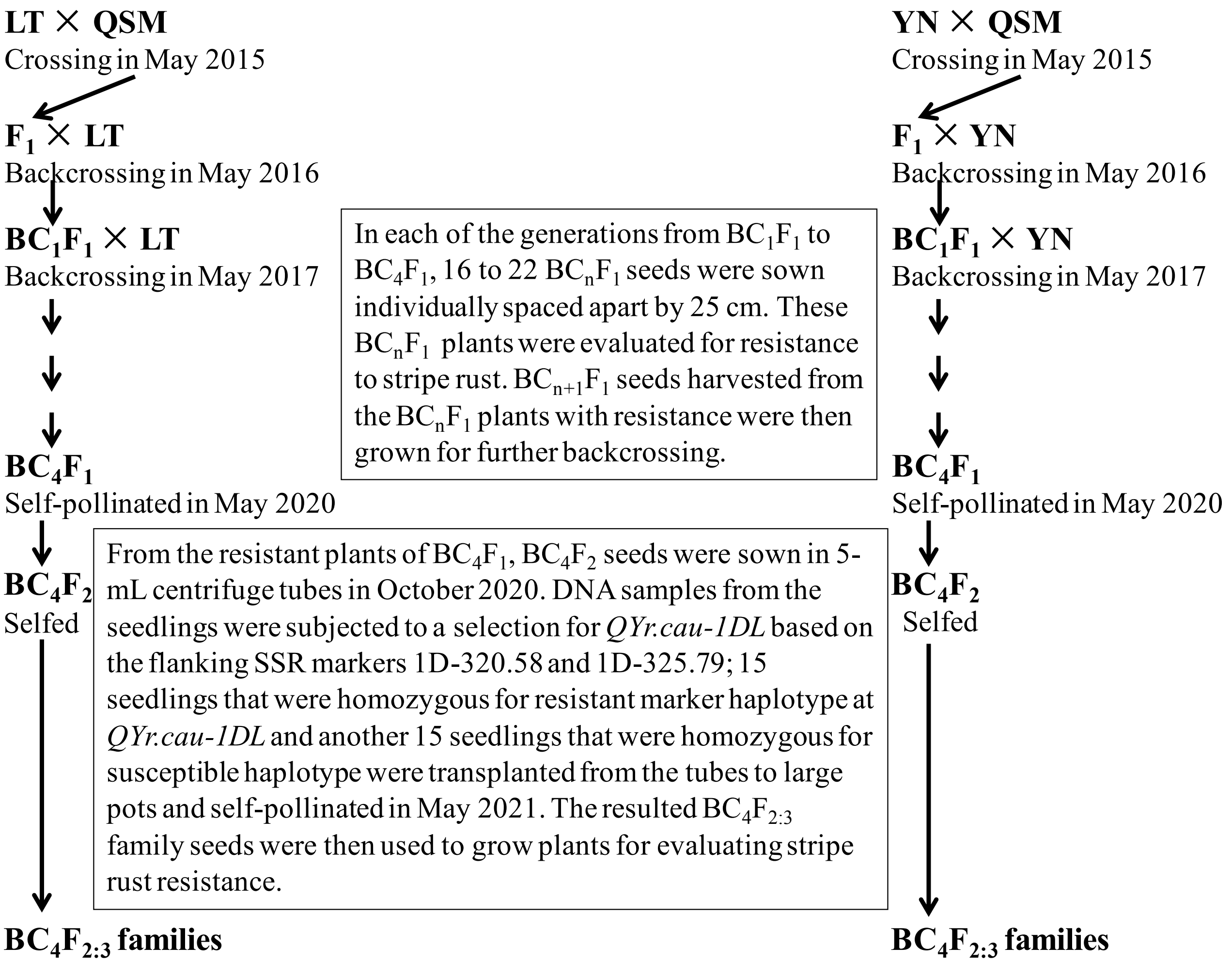

BC_4F_2 seeds of LT × QSM were sown, in October 2020, in 5-mL centrifuge tubes filled with moist soil with a single germinated seed in each tube. These seedlings were maintained in growth chambers at 5±2°C with a relative humidity higher than 70%. DNA samples extracted from these seedlings were subjected to a selection for *QYr.cau-1DL* based on the SSR markers 1D-320.58 and 1D-325.79 that delimited *QYr.cau-1DL* to a 0.5 cM interval (Figure 2B). PCR amplifications with QSM DNA as template produced a 232 bp amplicon at the marker locus 1D-320.58 and a 290 bp amplicon at 1D-325.79 (Figure 3). These two amplicons constitutes the marker haplotype that represents the resistant allele of *QYr.cau-1DL*. LT showed a 238 bp amplicon at 1D-320.58 and a 296 bp amplicon at 1D-325.80, a susceptible marker haplotype. This selection identified two marker groups, namely, 15 BC_4F_2 seedlings in homozygous state for resistant haplotype (R-group) and another 15 BC_4F_2 seedlings in homozygous state for susceptible haplotype (S-group). In the same way, R-group and S-group were selected from YN × QSM (YN showed the same susceptible haplotype as LT; Figure 3). Seedlings (60 ones in total) of the four groups were transplanted from the centrifuge tubes into large pots where they developed to adult plants and their spikes were bagged for self-pollination in May 2021. The resulted $BC_4F_{2:3}$ family seeds were grown subsequently for evaluating stripe rust resistance.

(2) $F_{2:3}$ families of RL6058 × QSM

To validate the effectiveness of *QYr.cau-1DL* in a genetic background of spring habit, and also to examine combination effect of *QYr.cau-1DL* with *Yr18*, RL6058 (a carrier of *Yr18*) plants were pollinated with QSM pollens in May 2019. F_2 seeds from the RL6058 × QSM F_1 plants were sown in 5-mL centrifuge tubes as described above. These F_2 seedlings were subjected to two selections using the primers as shown below.

Allele	Primer (Sequence from 5´ to 3´)	Annealing temperature (°C)	Expected amplicon size (bp)	Reference
vrn-A1	VRN1AF (gaaaggaaaaattctgctcg)	60	734	Yan et al. (2004)
	VRN1-INT1R (gcaggaaatcgaaatcgaag)			
Yr18[a]	L34SPF(gggagcattatttttttccatcatg)	58	751/523	Lagudah et al. (2009)
	L34DINT13R2(actttcctgaaaataatacaagca)			
	L34DINT9F(ttgatgaaaccagtttttttcta)			
	L34MINUSR(gccatttaacataatcatgatgga)			

[a] *A multiplex reaction was performed using the primer combinations L34SPF/L34DINT13R2 and L34DINT9F/L34MINUSR, which amplified a 751 bp fragment (resistant allele) and a 523 bp fragment (susceptible allele), providing a co-dominant marker (cssfr5)*

The first selection was conducted to establish a genetic background of spring habit. Our pilot experiments indicated that the F_2 population of RL6058 (spring habit) × QSM (winter habit) was segregating at *Vrn-A1* locus and monomorphic at the other *Vrn* loci including *Vrn-B1*, *Vrn-D1* and *Vrn-B3*. Using RL6058 DNA as template, PCR amplification with the primers for *vrn-A1* produced a 734 bp band that represents spring habit. F_2 seedlings with 734 bp band in homozygous state for *vrn-A1* were identified, and then were subjected to the second selection.

The second selection was based on the aforementioned SSR markers of *QYr.cau-1DL* and a marker (*cssfr5*) that is diagnostic of *Yr18* (Lagudah et al. 2009). PCR amplifications with QSM DNA as template illustrated a resistant haplotype (i.e., a 232 bp band combined with a 290 bp band) of *QYr.cau-1DL* and a susceptible allele (523 bp band) of *Yr18*, in contrast to the susceptible haplotype (a 223 bp combined with a 296 bp) and the resistant allele (a 751 bp) shown by RL6058 (Figure 3). PCR amplifications with the F_2 DNA samples as templates identified four marker groups, namely, the F_2 seedlings in homozygous state for the resistant haplotype at *QYr.cau-1DL* and the resistant allele at *Yr18* (*QYr.cau-1DL+Yr18*), resistant haplotype at *QYr.cau-1DL* but susceptible allele at *Yr18* (*QYr.cau-1DL* alone), susceptible haplotype at *QYr.cau-1DL* but resistant allele at *Yr18* (*Yr18* alone), and susceptible haplotype at *QYr.cau-1DL* and susceptible allele at *Yr18* (*None*; susceptible

control). The 80 F_2 seedlings (20 ones each group) were transplanted from the centrifuge tubes into large pots as described above. $F_{2:3}$ family seeds from each of the 80 F_2 plants were subsequently grown for evaluating stripe rust resistance.

References

Lagudah, E. S., Krattinger, S. G., Herrera-Foessel, S., Singh, R. P., Huerta-Espino, J., Spielmeyer, W., Brown-Guedira, G., Selter, L. L., and Keller, B. (2009). Gene-specific markers for the wheat gene *Lr34/Yr18/Pm38* which confers resistance to multiple fungal pathogens. *Theor. Appl. Genet.* 119, 889–898.

Yan, L., Helguera, M., Kato, K., Fukuyama, S., Sherman, J., and Dubcovsky, J. (2004). Allelic variation at the VRN-1 promoter region in polyploid wheat. *Theor. Appl. Genet.* 109, 677–1686.

Supplementary File 2 | Typical stripe rust symptoms on wheat varieties Qishanmai (QSM), Suwon11 (SW) and Reichersberg 42 (RC) (a carrier of *Yr25* and *Yr7*) infected with mixed urediniospores of *Pst* races CYR32 and CYR34.

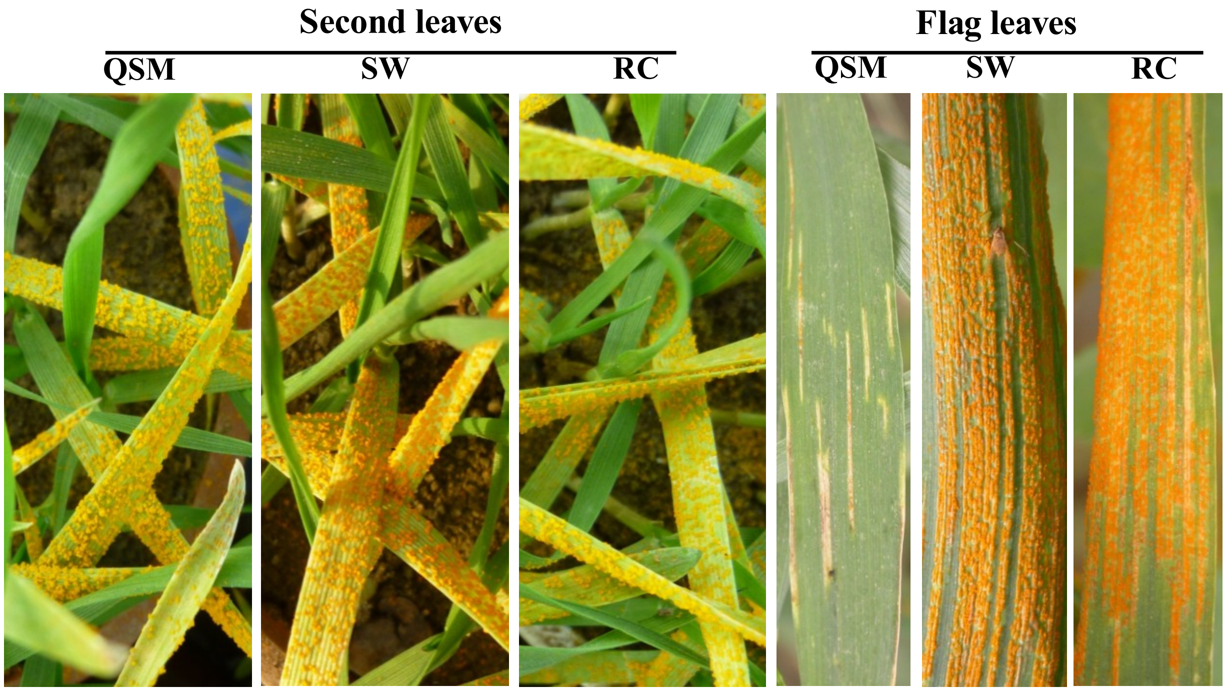

Supplement: Supplementary file 1 [file DataSheet_1.pdf]
